# Tackling the re-emergence of wheat stem rust in Western Europe

**DOI:** 10.1038/s42003-019-0294-9

**Published:** 2019-02-04

**Authors:** Diane G. O. Saunders, Zacharias A. Pretorius, Mogens S. Hovmøller

**Affiliations:** 1 John Innes Centre, Norwich Research Park, Norwich, NR4 7UH UK; 2 University of the Free State, Bloemfontein, 9301 South Africa; 3 Aarhus University, Flakkebjerg, Slagelse, 4200 Denmark

## Abstract

In our recent *Communications Biology* article, we reported the first occurrence of wheat stem rust in the UK in nearly six decades. An increased incidence of wheat stem rust in Western Europe, caused by the fungus *Puccinia graminis* f. sp. *tritici*, could signify the return of this formidable foe. As pathologists fight back against this devastating disease we outline the continuing research and strategies being employed to bridle its onslaught.

## Abstract (web summary)

Saunders et al. discuss the latest research and strategies used to control wheat stem rust in Western Europe following their report of its recurrence in the UK for the first time in almost 60 years. In their Comment, they hope to build on their previous work to drive innovation in disease management.

## First report in decades of a forgotten enemy in Western Europe

Wheat stem rust, caused by the fungus *Puccinia graminis* f. sp. *tritici*, has been associated with crop failures and famine throughout history. As early as 700 B.C., red animals were sacrificed to the rust god Robigus by the Romans each spring in hopes of protection for their grain fields from the reddish/brown rust spores^1^. Despite the virulence of the pathogen, a global community of plant pathologists and wheat breeders achieved the almost impossible: They prevented any significant stem rust epidemics in the major wheat growing areas worldwide in the last half of the twentieth century^2^, including Western Europe. Then, in 2013, pathologists in Germany raised the alarm about the re-emergence of that previously vanquished foe^3^.

Plant pathologists provided a fundamental understanding of the pathogen’s biology that informed disease management strategies^2^ while breeders actively bred stem rust resistance into wheat cultivars during the Green Revolution. Unfortunately, the landscape changed dramatically in 1998, when a new highly virulent race of wheat stem rust emerged in Uganda. The infamous Ug99 race^4^ rendered up to 80% of the world’s wheat varieties vulnerable to stem rust^5^. As expected from the transboundary nature of many rust fungi, Ug99 and its variants have now spread across East and Southern Africa and into the Middle East^6^. In response, the Borlaug Global Rust Initiative (BGRI) was created^7^, and research in wheat rust epidemiology and resistance breeding intensified, resulting in the release of multiple Ug99-resistant wheat varieties. Even so, these rapidly shifting events are a perilous reminder of the threat stem rust can pose to global food security^8^.

The 2013 regional epidemic of wheat stem rust in Germany was followed by a series of sporadic infections in countries including Denmark, Sweden and the UK^6^. There were few enough plants infected in these initial cases that pathologists saw this as an early warning—by contrast, average yield losses reached around fifty percent in 1955 during the last severe stem rust epidemic in England^9^. After these “early warning” infections, a much larger wheat stem rust outbreak in Sicily in 2016 affected thousands of hectares of both durum and bread wheat^10^. Also in 2015–2016, unprecedented epidemics of stem rust hit between one to two million hectares of spring wheat in Western Siberia, causing average yield losses of 30–40% on a regional scale^6,11^, and in 2017, a new outbreak of stem rust on late-maturing wheat and barley in the central part of Sweden was reported^12^.

Faced with these increasing episodes of stem rust—along with exotic incursions of aggressive new races of yellow rust, another wheat-damaging rust disease—pathologists and breeders across Europe rallied to form RustWatch, a consortium led by the Global Rust Reference Center at Aarhus University in Denmark^13^. The consortium’s main aim is to develop an early-warning system for wheat rusts by sharing facilities, knowledge, expertise, and results from national rust diagnostic laboratories and breeding programs throughout Europe. Trials involving new tools to assist rust resistance breeding and disease management practices in wheat are also planned during the 4-year project that was launched in 2018.

## Could wheat stem rust re-establish in the UK?

In light of recent events across Europe, we initiated a study in the UK bringing together the expertise of researchers across 14 countries to evaluate the risk of wheat stem rust re-establishment and its subsequent potential threat to UK wheat production. In this study, published in *Communications Biology*^14^, we uncovered a severe lack of resistance to the pathogen in current UK wheat varieties. More than 80% of the 57 varieties tested were highly susceptible to infection by an isolate of race TKTTF, which was prevalent in the 2013 outbreak in Germany and was detected in the UK and Denmark in the same year^14^. Following this study, we began additional analysis of a selection of UK wheat varieties in a stem rust-conducive area of South Africa. Preliminary results confirm that little resistance is present in the current European germplasm at either the seedling or adult plant stage. This unsettling conclusion is further supported by our recent analysis of a selection of Scandinavian wheat and barley varieties, which were found to have very little resistance to stem rust at the seedling stage^15^.

As stem rust primarily favours warmer conditions for infection, it is important to consider the influence of climatic change in potentially supporting stem rust re-establishment. Indeed, our UK study reported that climatic conditions over the past 25 years have become increasingly conducive to stem rust infection^14^. As summer temperatures now often occur earlier in the growing season, the earlier-maturing wheat varieties that had previously been able to avoid inoculum build-up could now be at risk. That was certainly the case in 2013 in Germany, where wet winter conditions delayed winter wheat development and earlier summer temperatures then favored stem rust infection^3^. Working closely with breeding companies, we are proactively searching for sources of resistance in current European germplasm that can be called upon if or when stem rust becomes re-established.

## The role of *Berberis* spp. in the stem rust life cycle

Each year, the asexual urediniospores of wheat stem rust can enter temporal zones (such as Western Europe) anew via wind transmission, traveling up to hundreds of kilometres from their origin in regions with milder continental climates^9^. However, stem rust also has the ability to survive between crop seasons in temporal zones through the production of hardy overwintering teliospores that can form on rust-infected plant debris. These teliospores germinate in the spring, producing basidiospores that can infect the pathogen’s alternate host, common barberry (*Berberis vulgaris*) and other *Berberis* species including many formerly placed in *Mahonia*. Once on the alternate host, the pathogen may complete sexual reproduction, leading to the emergence of novel genotypes and races^16^.

This role of *Berberis* species in the wheat stem rust lifecycle prompted legislation and exclusion campaigns to eradicate them across many countries in Europe and North America over the past century, with a particular focus on common barberry, which is highly susceptible to stem rust infection^17^. It was hoped that removing common barberry from wheat-growing areas would prolong the life of rust-resistant wheat varieties. In England and Scandinavia, the removal of common barberry actually began much earlier, in the eighteenth century when “English farmers complained that black stem rust ruined crops growing near barberry bushes. So, they showed their good sense by destroying the bushes”^18,19^. These movements were enormously successful in breaking the disease cycle and driving wheat stem rust to near extinction in Western Europe.

Unfortunately, legislation to restrict the planting of common barberry has long since lapsed, and the popular hedgerow shrub is again increasing in prevalence. Barberry has also more recently been shown to be part of the epidemiology of yellow rust in China^20,21^, a disease which infects cereal crops including wheat every year across Europe. At least three incursions of exotic yellow rust races have occurred in Europe in the past ten years, and although the pathogen is not currently known to undergo sexual reproduction in this region, the unusually high quantities of teliospores produced by these new races^22^ could expedite infection as common barberry becomes increasingly prevalent. Worryingly, we recently found that barberry bushes that occur close to cereal crops in the UK and Scandinavia can harbor forms of stem rust that are capable of infecting particularly barley and oat varieties in the laboratory environment^14^. Furthermore, the 2017 outbreak of stem rust in Sweden occurred in an area where common barberry has re-established, and our colleagues in Sweden have just identified a highly diverse sexual population of wheat stem rust in the area, likely derived from common barberry^12^.

These discoveries have heightened the awareness of the role of the alternate host in the disease cycle and underscore the need to understand the threat barberry could pose as an infection reservoir for rust diseases on cereals and grasses. One of our current studies on common barberry aims to survey rust fungal species on common barberry in European countries under the aegis of RustWatch. In another ongoing study, we are surveying rust fungal species as well as mapping the whereabouts of common barberry shrubs in the UK, to highlight those in close proximity to cereal crops that could be at high risk of harboring cereal rust and therefore should be carefully monitored.

## Balancing species conservation with cereal rust resilience

Common barberry was introduced to Europe from Asia in the Middle Ages and entered Western Europe in the seventeenth century as a new resource rich in vitamin C and as an efficient hedge shrub to prevent forest animals from destroying agricultural crops^23,24^. Common barberry also provides an essential habitat for various wildlife. For instance, the larvae of the barberry carpet moth (*Pareulype berberata*) are reliant on common barberry as their only known food source^25^. Accordingly, the mass removal of common barberry across the UK in the nineteenth and twentieth centuries, while vital in the battle against wheat stem rust, had a knock-on impact on biodiversity and drove the barberry carpet moth to near extinction.

The barberry carpet moth remains at grave risk of becoming another victim of UK species loss. Given the reduction in barberry plantings, the limited number of local barberry carpet moth populations are small and therefore vulnerable to local extinctions. The moth is bivoltine, producing two generations per year with larvae occurring in June/July and August/September. The pupae overwinter in leaf litter below the barberry bushes before emergence in May/June or August^26^. A careful balance will be required to improve the carpet moth habitat, i.e., the common barberry that has been present in Europe for centuries^23^, without promoting a virulent resurgence of UK wheat stem rust outbreaks. To address this conundrum, we are working closely with the farming community and conservation groups in the UK to develop new knowledge that can be used to guide habitat enhancement programs whilst also safeguarding UK wheat crops from an escalation in cereal rust diversity.

## Concluding remarks

As we enter an era in which wheat stem rust appears increasingly likely to re-emerge across Europe, rust pathologists are once again at the forefront of the fight back against this formidable foe, using the latest knowledge of the pathogen’s biology and modern breeding strategies. However, we worry that the ever-decreasing numbers of traditional plant pathologists will be insufficient to prevent wheat stem rust resurgence, especially alongside the complacency in the wider community arising from so many decades free from stem rust epidemics.

Ensuring long-lasting resilience against these pressing threats to crop productivity requires investment in training the next generation of plant pathologists and plant breeders to impart the skills and wisdom of a community that has time and again wrestled wheat stem rust into check. We also need to continue efforts to improve our basic understanding of the pathogen’s biology and to drive innovation in plant breeding and disease management that could sow the seed for the next green revolution.
